# Head Nurse Digital Leadership and Staff Nurse–GenAI Collaboration in Chinese Hospitals: The Mediating Role of Digital Self‐Efficacy and the Moderating Role of Nurse Perceived Job Autonomy

**DOI:** 10.1155/jonm/8889364

**Published:** 2026-06-25

**Authors:** Qian Wu

**Affiliations:** ^1^ Department of Nursing, North Anhui Health Vocational College, Suzhou, Anhui, 234000, China

**Keywords:** digital leadership, digital self-efficacy, job autonomy, nurse, nurse–GenAI collaboration

## Abstract

**Aims:**

To examine how head nurse digital leadership is associated with staff nurse–generative artificial intelligence (GenAI) collaboration, specifically exploring the mediating role of digital self‐efficacy and the moderating role of nurse perceived job autonomy.

**Background:**

GenAI is shifting nursing practice from solo task execution to active nurse–AI collaboration. While digital leadership is theorised to facilitate this transition, the specific mechanisms and boundary conditions, particularly in the context of Chinese tertiary hospitals, remain insufficiently explored.

**Methods:**

A time‐lagged study design was employed, collecting data from 336 frontline nurses across 18 tertiary hospitals in China. The moderated mediation model was tested using PROCESS Model 4 and PROCESS Model 7 with 5000 bootstrap resamples.

**Results:**

Digital leadership was positively associated with nurse–GenAI collaboration. Digital self‐efficacy served as a critical mediator in this relationship. Furthermore, perceived job autonomy significantly moderated the relationship between digital leadership and digital self‐efficacy. The indirect effect of leadership on collaboration through self‐efficacy was also significant when nurses reported higher levels of job autonomy.

**Conclusion:**

Head nurses who demonstrate strong digital leadership are associated with more effective GenAI collaboration, potentially by nurturing nurses’ digital confidence. However, the strength of this association appears contingent upon the structural resource of job autonomy, which may afford nurses greater freedom to experiment with and integrate AI tools into clinical practice.

**Implications for Nursing Management:**

Nursing leaders should move beyond top–down AI implementation towards distributed leadership models that prioritise the development of nurses’ digital self‐efficacy. To maximise the benefits of AI adoption, hospital administrators must foster autonomous work environments that empower nurses to act as proactive partners with technology, ultimately enhancing workflow efficiency and clinical resilience in high‐workload settings.

## 1. Introduction

The integration of generative artificial intelligence (GenAI) into healthcare is rapidly transforming the delivery of care, shifting nursing practice from solo task execution towards active human–AI collaboration [1]. As frontline providers, nurses play a pivotal role in utilising AI to enhance patient outcomes and support complex clinical decision‐making [2]. However, the success of AI implementation is a sociotechnical challenge that hinges on the successful coalignment of organisational, human and technical systems [3]. Digital leadership has emerged as a critical driver in this transition, as nursing leaders are increasingly tasked with fostering AI literacy, ensuring ethical oversight and managing shifting workforce dynamics [4]. In the context of high‐workload clinical environments, such leadership is essential to guide nursing staff through AI transitions by prioritising organisational readiness and adopting strategies that integrate technical education with collaborative change [5].

Despite the growing presence of GenAI in healthcare, many nurses remain underprepared for meaningful engagement with these tools, often leading to rational resistance driven by concerns over workflow disruption and professional autonomy [6]. While the literature cautions against a purely technological deterministic view, little is known about the specific mechanisms through which digital leadership translates into daily GenAI collaboration [7]. Specifically, there is a lack of evidence regarding how leaders move beyond traditional top–down implementation models to build the necessary psychological resources, such as digital self‐efficacy, that empower nurses to work confidently alongside AI [8]. Furthermore, the boundary conditions, such as perceived job autonomy, that may amplify or hinder the influence of leadership on nurse engagement in complex sociotechnical systems remain largely unexplored.

This research endeavours to shed light on these critical relationships within the context of Chinese tertiary hospitals. By examining the association between head nurses’ digital leadership and staff nurses’ GenAI collaboration, with digital self‐efficacy as a proposed mediator and perceived job autonomy as a proposed moderator, this study aims to contribute valuable insights to the nursing management literature and offer practical implications for healthcare organisations navigating AI integration. Specifically, the study addresses the following research questions: (1) How is digital leadership associated with GenAI collaboration within nursing organisations? (2) What is the potential mediating role of nurse digital self‐efficacy in the relationship between head nurse digital leadership and nurse–GenAI collaboration? (3) Does the nurse perceived job autonomy moderate the relationship between digital leadership and digital self‐efficacy? (4) Is the indirect association between head nurse digital leadership and nurse–GenAI collaboration through digital self‐efficacy conditional on nurse perceived job autonomy?

Given that the effects of leadership on technology adoption are rarely uniform, a simple mediation model through digital self‐efficacy is insufficient to capture the conditional mechanisms operating in high‐workload clinical environments. Prior literature indicates that the leadership–efficacy relationship is strengthened or weakened by contextual resources such as job autonomy [9, 10]. Therefore, a moderated mediation approach was adopted to examine whether the indirect effect of digital leadership on nurse–GenAI collaboration via digital self‐efficacy is conditional on perceived job autonomy. This design provides a more nuanced understanding of the boundary conditions under which digital leadership translates into effective human–AI partnership.

These findings contribute to the nursing management literature by offering evidence‐informed insights for moving towards adaptive, distributed leadership models that treat nurses as true partners with AI [11, 12]. By identifying digital self‐efficacy as a potential mediator, this research highlights how leadership may be associated with reduced underuse or misuse of AI through the fostering of standardised AI competencies and ethical scrutiny. Furthermore, by examining the moderating role of job autonomy, this study offers insights into how hospital administrators may jointly optimise technical systems with nursing practices to support workflow efficiency and resilience. Ultimately, this work addresses the gap between theory and practice, informing leadership strategies that may sustain equitable and resilient AI‐driven healthcare systems.

China’s healthcare sector has undergone rapid growth and modernisation in recent years, driven by substantial government investment in facilities, advanced technologies and workforce training to enhance service delivery [13]. The system has shifted from predominantly state‐funded to include greater private‐sector participation, promoting competition and innovation [14]. Tertiary hospitals, as leading institutions, are at the forefront of these developments, particularly in nursing care, with both public and private facilities committed to high‐quality services [15].

Advanced technologies, including GenAI, play a central role in healthcare reforms. Tertiary hospitals nationwide have implemented sophisticated IT systems to improve patient records, administrative efficiency, diagnostics and therapeutics [16]. GenAI applications, such as clinical decision support and predictive analytics, hold strong potential to transform nursing practice [17]. However, integrating GenAI into nursing roles introduces significant changes to workflows and workforce resilience [18]. Successful adoption depends heavily on nursing competencies, supported by extensive government‐funded training programmes [19]. Digital leadership by head nurses is therefore considered important for advancing innovation and addressing challenges, with job autonomy proposed as a key boundary condition in the pathway connecting leadership, digital self‐efficacy and nurse–GenAI collaboration [20].

## 2. Theory and Hypotheses Development

### 2.1. COR Theory

COR theory posits that individuals are motivated to obtain, retain, foster and protect those things they value, which are defined as resources [21, 22]. These resources can be categorised into objects, personal characteristics, conditions and energies. A central tenet of the theory is the resource caravan, which suggests that resources do not exist in isolation but rather accumulate and support one another [22]. When individuals possess a strong pool of resources, they are better equipped to handle job demands and are more likely to seek out further resource gains [21]. Conversely, the theory also highlights the loss spiral, where individuals lacking resources are more vulnerable to further loss, particularly in high‐pressure environments like healthcare, where workload and staffing shortages are prevalent [22].

**FIGURE 1:**
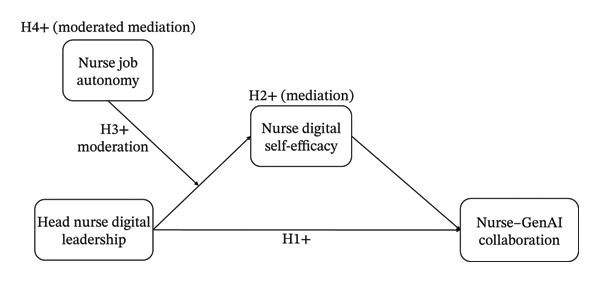
Conceptual framework. Source: author’s own work.

In the framework of this study (Figure 1), COR theory provides a lens through which digital leadership may act as a contextual resource that initiates a resource caravan for frontline nurses. Head nurses exhibit digital leadership supply condition resources (organisational support and vision) that may facilitate nurses’ accumulation of personal resources such as digital self‐efficacy. High job autonomy functions as an additional condition resource that may buffer against loss spirals, potentially reducing the depletion of efficacy that can occur when nurses face rigid structures during technological disruption, and may accelerate resource‐gain cycles. Conversely, constrained autonomy risks initiating loss spirals, whereby even strong leadership may be insufficient to build confidence, potentially contributing to reduced GenAI collaboration and heightened rational resistance. This dynamic interplay between resource gain and loss underscores the sociotechnical reality of AI integration in nursing.

### 2.2. Head Nurse Digital Leadership and Nurse–GenAI Collaboration

Digital leadership involves the ability of a leader to articulate a clear vision for digital transformation and support the development of a digital mindset [23] among staff. In the context of GenAI, head nurses serve as the primary facilitators who move the team beyond top–down implementation by fostering AI literacy and engagement. By modelling AI proficiency and promoting ethical oversight, these leaders lower the barriers to entry, enabling nurses to transition from solo task execution to an active partnership with AI tools [24]. This leadership creates a sociotechnical environment where GenAI is not viewed as a threat, but as a collaborative tool for information identification and decision support [25].

From the perspective of COR theory, digital leadership functions as a critical contextual resource. By providing encouragement and a clear digital vision, the head nurse supplies condition resources that may reduce the perceived risk of technological change [26]. According to the principle of resource investment, nurses are more likely to invest their time and effort into complex GenAI collaboration when supported by a resource‐rich leadership environment. This proactive leadership may help buffer the resource loss often associated with technological disruption and is associated with greater engagement in collaborative behaviours that enhance clinical workflow efficiency.

 H1: Head nurse digital leadership is positively associated with nurse–GenAI collaboration.

### 2.3. Nurse Digital Self‐Efficacy as a Mediator

In clinical nursing environments, head nurse digital leadership functions as a specialised contextual resource that directly shapes how frontline staff appraise their work demands [27]. Unlike generalised corporate vision‐setting, digital leadership in nursing is expressed through concrete behavioural actions. These actions include modelling algorithmic verification, framing GenAI as a decision‐support tool rather than a clinical replacement and actively reducing technological anxieties [28]. Drawing on COR theory, these targeted inputs construct a localised resource caravan that provides nurses with the informational and emotional resources needed to develop nurse‐specific digital self‐efficacy. Digital self‐efficacy refers to a nurse’s task‐specific confidence to utilise, critically appraise and correct emerging digital technologies in clinical practice [10, 29].

When head nurses demonstrate digital leadership competencies such as openly discussing GenAI capabilities, establishing clear boundaries for algorithmic accountability, and cultivating psychological safety around technological experimentation [5], they may provide frontline nurses with vicarious modelling and social persuasion [4]. Both are well‐established antecedents of self‐efficacy development. For example, when nurses observe their leaders successfully verify GenAI‐generated care plans against established professional standards, cognitive apprehension about algorithmic errors or workflow disruption may diminish [2]. This form of supportive supervision may act as an environmental resource, potentially facilitating the conversion of external technological demands into internal personal resources. As digital self‐efficacy develops, frontline nurses may be less likely to approach GenAI with defensive resistance. Instead, they may build the clinical confidence necessary to engage in active nurse–GenAI collaboration, integrating technology into complex decision‐making while maintaining professional oversight [30].

COR theory suggests that resources do not exist in isolation but form resource caravans where one resource may facilitate the development of another. In this study, the contextual resource of digital leadership is theorised to facilitate the acquisition of a personal characteristic resource: digital self‐efficacy. This accumulation of resources may enable nurses to engage in more advanced resource‐generating behaviours, such as active human–AI collaboration. Digital self‐efficacy is posited to act as the bridge in this caravan, potentially translating the leader’s external support into the nurse’s internal motivation to work as a true partner with AI.

 H2: Nurse digital self‐efficacy mediates the link between head nurse digital leadership and nurse–GenAI collaboration.

### 2.4. Nurse Job Autonomy as a Moderator

In the institutional environment of Chinese tertiary hospitals, job autonomy operates within a highly centralised organisational structure characterised by distinct hierarchical lines, high‐power distance, and collectivist operational norms [31]. In this context, job autonomy does not imply a complete detachment from institutional oversight. Instead, it manifests as a localised, discretionary resource: the explicitly permitted latitude for frontline nurses to exercise clinical judgement, alter workflow sequences and independently verify clinical data at the bedside [32]. Because institutional protocols in tertiary facilities are traditionally top–down [33], the level of autonomy perceived by a frontline nurse is heavily dependent on their head nurse. The head nurse serves as an institutional gatekeeper who can either rigidly enforce administrative uniformity or deliberately protect spaces for staff experimentation with digital tools [34].

Under COR theory, this localised job autonomy functions as an essential contextual condition resource that may shape the utility of leadership inputs. When head nurses exhibit strong digital leadership within a high‐autonomy environment, the structural permission to act may reinforce the leader’s digital vision [10, 35]. This may afford nurses the operational flexibility to integrate GenAI tools into their daily schedules, such as using AI for drafting nursing care plans or analysing patient data, without fearing immediate administrative penalties for minor process deviations. This synergy may accelerate resource accumulation, potentially facilitating the translation of the head nurse’s support into personal digital self‐efficacy.

Conversely, if job autonomy is constrained by rigid top–down structures, a low‐autonomy environment may contribute to a resource loss spiral. Even if a head nurse demonstrates exemplary digital leadership, their supportive signals may be weakened if nurses face severe penalties for deviating from strict, nondigital workflow mandates. Frontline staff may find it difficult to translate leadership support into personal confidence when they lack the practical freedom to use the technology [9]. Thus, in Chinese healthcare environments, perceived job autonomy serves as a critical boundary resource. It may provide the structural security necessary for frontline nurses to internalise e‐leadership initiatives and develop digital self‐efficacy.

 H3: Nurse job autonomy moderates the positive relationship between head nurse digital leadership and nurse digital self‐efficacy, such that the relationship is stronger (vs. weaker) when nurse job autonomy is high (vs. low).

### 2.5. Moderated Mediation

The indirect association between digital leadership and GenAI collaboration through digital self‐efficacy is expected to be contingent upon the level of job autonomy. High autonomy may provide the structural freedom that facilitates nurses’ translation of leadership support into self‐efficacy and, ultimately, into active collaboration behaviours [9, 29]. In high‐autonomy environments, digital leadership may more effectively support the development of confidence required for nurses to treat GenAI as a true partner. Without such autonomy, the mediated pathway may be attenuated. Thus:

 H4: Nurse job autonomy moderates the indirect relationship (via nurse digital self‐efficacy) between head nurse digital leadership and nurse–GenAI collaboration such that the indirect effect is stronger (vs. weaker) when nurse job autonomy is high (vs. low).

## 3. Methods

### 3.1. Sample and Data Collection

Hospitals were purposively selected from six geographic regions (North, Northeast, East, South, Southwest, Northwest) using national healthcare databases and nursing association lists to ensure geographic and institutional diversity; within each region, three tertiary hospitals were chosen (two public, one private) based on documented GenAI adoption. Eligible participants were full‐time frontline staff nurses and their heads with at least six months of GenAI experience in clinical practice, as this duration has been shown in prior studies to allow sufficient familiarity for reliable self‐reporting of technology‐related behaviours [29]. Inclusion criteria: registered nurses employed for ≥ 6 months in the selected hospitals with documented GenAI use. Exclusion criteria: part‐time staff or nurses without GenAI exposure. A two‐stage cluster sampling approach was used: Hospitals were clustered by region, and then, nurses and their heads were recruited via convenience sampling within hospital nursing departments. Surveys were administered in Mandarin (back‐translated) and were voluntary and anonymous.

To minimise potential common method variance (CMV) inherent in self‐reported survey data, this study implemented strict procedural and statistical remedies [36]. Procedurally, the data collection employed a two‐wave time‐lagged design with a temporal separation of 2 weeks; additionally, staff nurses were asked to rate their head nurses’ digital leadership behaviour rather than self‐rate. This design effectively disrupts the cognitive anchors responsible for consistency artefacts, transient mood states and evaluation apprehension. At Wave 1 (October–November 2025), participants rated the independent variable (head nurse digital leadership) and the moderator (nurse job autonomy). At Wave 2 (December 2025–January 2026), they rated the mediator (nurse digital self‐efficacy) and the dependent variable (nurse–GenAI collaboration). Prior to data collection, informed consent was obtained from all participating head nurses and staff nurses. Participants were assured of data confidentiality, anonymity, and their right to withdraw from the study at any point without any negative consequences. The survey items were counterbalanced across sections, and the wording was refined during back‐translation to eliminate ambiguous or leading phrasing. The final response rate was 78%, yielding 336 complete matched responses.

Statistically, Harman’s single‐factor test was used to verify the efficacy of these procedural controls. The test was executed by entering all continuous manifest variables into an unrotated exploratory factor analysis. The results revealed that the first factor accounted for only 24.3% of the total variance, which falls well below the standard 50% threshold. This indicates that no single dominant factor accounts for the majority of the variance in our data.

### 3.2. Measures

All constructs were assessed using established and pretested but slightly adapted scales with a 5‐point Likert‐type response format (1 = *strongly disagree* to 5 = *strongly agree*). All scales were slightly adapted for the GenAI nursing context following standard forward‐ and back‐translation procedures. A pilot study with 30 nurses confirmed face validity and internal consistency (Cronbach’s *α* > 0.80 for all scales). Items were refined for relevance to GenAI in the Chinese nursing context while preserving original meaning and psychometric properties. Head nurse digital leadership was measured with a 5‐item scale [34] capturing both visionary (e.g., communicating a clear digital/AI future) and supportive behaviours relevant to clinical nursing (sample item: ‘My head nurse communicates a clear digital and AI‐enabled vision for the future of nursing care’). Nurse Digital Self‐Efficacy (self‐rated) was assessed using an adapted 6‐item scale [37]. A sample item is ‘I am comfortable learning new digital and AI‐based technology.’ Nurse–GenAI collaboration was adapted [38] to reflect clinical realities such as co‐decision‐making and verification of AI outputs against professional judgement (sample item: ‘GenAI participates in my clinical decision‐making process (e.g., formulating nursing care plans)’). Job Autonomy (self‐rated) was measured using the three‐item decision‐making autonomy subscale from the Work Design Questionnaire (WDQ) [39]. Sample item: ‘The job gives me a chance to use my personal initiative or judgement in carrying out the work’. These adaptations preserved original meaning while aligning with the definition of collaboration as information identification and decision support in nursing practice [25].

## 4. Data Analysis

Data analysis was conducted in two stages using SPSS Version 30.0 and AMOS Version 28.0. First, confirmatory factor analysis (CFA) was performed in AMOS 28.0 to evaluate the validity and reliability of the measurement scales. A four‐factor measurement model (head nurse digital leadership, nurse digital self‐efficacy, nurse–GenAI collaboration and nurse job autonomy) was tested. Model fit was assessed using the chi‐square to degrees of freedom ratio (*X* ^2^/df), comparative fit index (CFI), Tucker–Lewis index (TLI) and root mean square error of approximation (RMSEA). Convergent validity was examined via factor loadings, average variance extracted (AVE) and composite reliability (CR). Discriminant validity was assessed using the Fornell–Larcker criterion. Second, hypotheses were tested using Hayes’ PROCESS macro (Version 4.2) in SPSS 30.0 with 5000 bootstrap resamples and bias‐corrected 95% confidence intervals. To examine the direct and indirect (mediating) effects, Model 4 was used. To test the moderating effect of job autonomy on the relationship between digital leadership and digital self‐efficacy, as well as the conditional indirect (moderated mediation) effect, Model 7 was employed. All continuous variables were mean‐centred prior to analysis to reduce multicollinearity and facilitate interpretation of interaction terms.

## 5. Results

The sample comprised 336 frontline nurses with a mean age of 39.94 years (SD = 6.39). Regarding gender, females represented (53%, *n* = 178) and males (47%, *n* = 158). Educationally, the sample was well‐distributed: 22.6% had secondary school qualifications, 19.6% junior college, 28.6% a bachelor’s degree and 29.2% a graduate degree. Mean tenure was 3.42 years (SD = 1.53), with the majority of nurses having 3–4 years of tenure (59.3%). Table 1 presents the descriptive statistics and correlations among study variables and constructs. The bivariate correlations were positive and significant, providing preliminary support for the hypothesised relationships. None of the control variables (age, gender, education, tenure) showed significant correlations with the outcome construct and was therefore excluded from further analyses to preserve model parsimony.

**TABLE 1:** Descriptive statistics and correlations.

Construct	Mean	SD	Age	Gender	Education	Tenure	HNDL	NDSE	NGAIC	NJA
Age	39.94	6.39								
Gender	—	—	0.08							
Education	2.64	1.13	−0.05	−0.06						
Tenure	3.42	1.53	0.02	0.02	0.09					
HNDL	4.18	0.61	−0.09	0.16^∗∗∗^	−0.01	−0.04	**0.786**			
NDSE	3.99	0.57	−0.01	0.07	−0.07	−0.01	0.27^∗∗∗^	**0.769**		
NGAIC	4.01	0.57	−0.06	0.001	−0.05	−0.09	0.29^∗∗∗^	0.37^∗∗∗^	**0.747**	
NJA	4.16	0.62	−0.08	0.08	−0.02	0.01	0.34^∗∗∗^	0.22^∗∗∗^	0.16^∗∗^	**0.892**

*Note: N* = 336. Bolded diagonal values represent the square root of the average variance extracted. Source: prepared by the authors based on results from the analysis.

Abbreviations: HNDL = head nurse digital leadership, NDSE = nurse digital self‐efficacy, NGAIC = nurse–GenAI collaboration, NJA = nurse job autonomy, SD = standard deviation.

^∗∗^ *p* < 0.01.

^∗∗∗^ *p* < 0.001.

### 5.1. Measurement Model Assessment

CFA was conducted using AMOS 28.0 to evaluate the validity and reliability of the measurement model. The four‐factor model (head nurse digital leadership, nurse digital self‐efficacy, nurse–GenAI collaboration and nurse job autonomy) demonstrated adequate fit: *X* ^2^/df = 2.229; CFI = 0.926; TLI = 0.912; and RMSEA = 0.078 (90% CI [0.073, 0.090]). These indices met or exceeded recommended thresholds (*X* ^2^/df < 3; CFI/TLI > 0.90; RMSEA < 0.08) [40].

Table 2 presents the results of convergent validity and reliability of the scales. Factor loadings were greater than 0.50, exceeding the 0.50 cut‐off for convergent validity [41]. CR values ranged from 0.771 to 0.874, surpassing 0.70, and AVE values ranged from 0.512 to 0.585, exceeding 0.50, confirming convergent validity [42]. Cronbach’s *α* values were also greater than 0.70, confirming adequate internal consistency [43]. Discriminant validity was confirmed using the Fornell–Larcker criterion. As shown in Table 1, the square root of each construct’s AVE (bolded diagonal values) exceeded its correlations with all other constructs [42].

**TABLE 2:** Convergent validity and reliability.

Construct	FL	AVE	CR	Cronbach’s *α*
*Head nurse digital leadership (HNDL)*		0.618	0.890	0.890
HNDL1	0.803			
HNDL2	0.764			
HNDL3	0.817			
HNDL4	0.727			
HNDL5	0.815			

*Nurse digital self-efficacy (NDSE)*		0.592	0.897	0.896
NDSE1	0.820			
NDSE2	0.755			
NDSE3	0.768			
NDSE4	0.752			
NDSE5	0.812			
NDSE6	0.703			

*Nurse–GenAI collaboration (NGAIC)*		0.559	0.863	0.861
NGAIC1	0.674			
NGAIC2	0.850			
NGAIC3	0.690			
NGAIC4	0.781			
NGAIC5	0.729			

*Nurse job autonomy (NJA)*		0.796	0.921	0.916
NJA1	0.946			
NJA2	0.812			
NJA3	0.913			

*Note:* Source: prepared by the authors based on results from the analysis.

Abbreviations: AVE = average variance extracted, CR = composite reliability, FL = factor loading.

### 5.2. Hypothesis Testing

**FIGURE 2:**
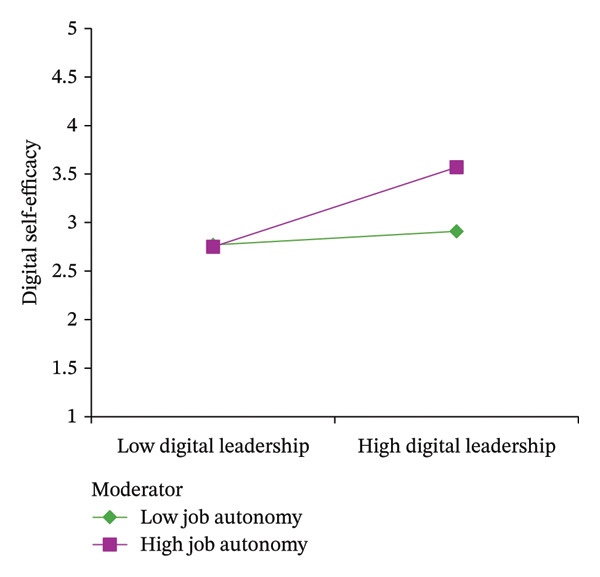
Nurse job autonomy strengthens the positive relationship between head nurse digital leadership and nurse digital self‐efficacy. Source: prepared by the authors based on results from the analysis.

Table 3 presents the hypothesis testing results. Hypotheses were tested in SPSS 30.0 using Hayes’ PROCESS macro, Model 4 for direct and indirect effects, and Model 7 for moderation and moderated mediation, with 5000 bootstrap resamples [44]. Bootstrapped confidence intervals (95% CI) were used to assess significance. Head nurse digital leadership was positively associated with nurse–GenAI collaboration (BootB = 0.19, *p* < 0.01, 95% BootCI [0.10, 0.28]), supporting H1. Nurse digital self‐efficacy accounted for a significant portion of this association between head nurse digital leadership and nurse–GenAI collaboration (Bootβ = 0.08, 95% BootCI [0.04, 0.13]), supporting H2. The direct effect remained significant, indicating partial indirect effects. Nurse job autonomy moderated the relationship between head nurse digital leadership and nurse digital self‐efficacy (Bootβ = 0.17, *p* < 0.01, 95% BootCI [0.05, 0.30]), supporting H3. Simple slope analysis depicted in Figure 2 revealed a stronger positive relationship at high job autonomy (Bootβ = 0.35, 95% BootCI [0.21, 0.49]) compared to low job autonomy (Bootβ = 0.14, 95% BootCI [0.02, 0.25]). Finally, the bias‐corrected confidence intervals for the index of moderated mediation excluded zero (Bootβ = 0.05, 95% BootCI [0.004, 0.12]), confirming that nurse job autonomy moderated the indirect association between head nurse digital leadership and nurse–GenAI collaboration via nurse digital self‐efficacy; thus, H4 was supported. The conditional indirect effect was larger at high job autonomy (Bootβ = 0.11, 95% BootCI [0.06, 0.18]) than at low job autonomy (Bootβ = 0.04, 95% BootCI [−0.01, 0.10]).

**TABLE 3:** Hypothesis testing.

Path/Effect	Boot estimate	BootSE	BootLLCI	BootULCI
*Direct effect*				
H1: HNDL ⟶ NGAIC	0.19^∗∗^	0.05	0.10	0.28

*Indirect effect*				
H2: HNDL ⟶ NDSE ⟶ NGAIC	0.08	0.02	0.04	0.13

*Conditional direct effect*				
H3: HNDL^∗^NJA ⟶ NDSE (interaction)	0.17^∗∗^	0.06	0.05	0.30
At low NJA (−1 SD)	0.14^∗^	0.06	0.02	0.25
At high NJA (+1 SD)	0.35^∗∗∗^	0.07	0.21	0.49

*Conditional indirect effects*				
At low NJA (−1 SD)	0.04	0.03	−0.01	0.10
At high NJA (+1 SD)	0.11	0.03	0.06	0.18

*Index of moderated mediation*				
HNDL^∗^NJA (moderated indirect effect)	0.05	0.03	0.004	0.12

*Note:* Model 4 = direct and mediation effects; Model 7 = moderation and moderated mediation effects. Source: prepared by the authors based on results from the analysis.

Abbreviations: HNDL = head nurse digital leadership, NDSE = nurse digital self‐efficacy, NGAIC = nurse–GenAI collaboration, NJA = nurse job autonomy.

^∗^ *p* < 0.05.

^∗∗^ *p* < 0.01.

^∗∗∗^ *p* < 0.001.

## 6. Discussion

The present study examined the role of head nurse digital leadership in its association with frontline nurse–GenAI collaboration in Chinese tertiary hospitals, with nurse digital self‐efficacy as a proposed mediator and nurse perceived job autonomy as a proposed moderator. The findings indicate that head nurse digital leadership is positively associated with nurse–GenAI collaboration, both directly and indirectly through nurse digital self‐efficacy. The results further show that nurse perceived job autonomy significantly moderates the relationship between head nurse digital leadership and nurse digital self‐efficacy, as well as the indirect effect of head nurse digital leadership on nurse–GenAI collaboration. These results align with the broader sociotechnical perspective on AI integration in nursing, where success depends not only on technological capabilities but also on human factors such as leadership, self‐confidence and contextual empowerment [3, 45].

This pattern is consistent with COR theory’s resource‐caravan and loss‐spiral mechanisms. Strong digital leadership may supply condition resources that facilitate the accumulation of personal efficacy; high autonomy may then buffer against loss spirals and support resource‐gain cycles, potentially affording nurses the opportunity to experiment with GenAI without fear of workflow disruption. In the hierarchical context of Chinese tertiary hospitals, where high‐power distance and collective decision‐making norms often constrain individual autonomy [16, 46], this conditional pattern highlights why leadership alone may be insufficient and why structural empowerment appears essential for sustained human–AI partnership. It should be noted that the concurrent measurement of digital self‐efficacy and GenAI collaboration at Wave 2 limits definitive causal inference regarding the mediation pathway; the observed indirect association is consistent with, but does not confirm, the proposed resource‐caravan process. Future three‐wave studies with temporally separated measurements of all constructs would provide stronger evidence for temporal precedence.

Consistent with prior research on digital transformation in healthcare, digital leadership appears as a meaningful correlate of adaptive human–AI partnerships, potentially operating through the provision of vision, encouragement, and role modelling for GenAI use [47]. The partial indirect association via digital self‐efficacy extends existing evidence that confidence in handling emerging technologies may serve as a bridge between leadership support and practical adoption. The moderating role of job autonomy further illustrates how perceived freedom in clinical decision‐making may strengthen the leadership–self‐efficacy pathway, potentially enabling nurses to invest more effectively in GenAI collaboration amid high‐workload environments. These patterns underscore the value of adaptive, distributed leadership to overcome rational resistance, build AI literacy and promote ethical, context‐specific GenAI integration in nursing [46, 48].

### 6.1. Theoretical Implications

This research contributes to the theoretical understanding of human–AI collaboration in nursing by integrating COR theory into a moderated mediation framework. The model illustrates how leadership may initiate resource caravans, how autonomy may buffer against loss spirals, and how self‐efficacy may serve as the pivotal personal resource that facilitates the translation of external support into collaborative behaviours [30], thus offering a theoretically grounded application of COR to GenAI contexts in nursing.

The Chinese healthcare context further enriches COR theory by highlighting how national cultural values, particularly high‐power distance and collectivistic orientations, may shape resource‐gain processes in technology adoption. In such settings, formal hierarchical structures may constrain nurses’ ability to act on leadership support, suggesting that perceived job autonomy may function as an important protective resource against loss spirals. This finding extends the generalisability of COR‐based models and underscores the importance of culturally sensitive approaches to digital leadership and work design in nursing.

By building on COR theory in GenAI contexts, the study advances sociotechnical theories of technology adoption in nursing [49], emphasising boundary conditions (autonomy) that explain variability in AI engagement. It also bridges digital leadership literature with self‐efficacy and autonomy theories, offering a nuanced model for why leadership effects on AI collaboration are not uniform but conditional on empowering work design elements.

### 6.2. Practical Implications

The results offer evidence‐informed guidance for nursing leaders and hospital administrators. Head nurses may wish to demonstrate visible digital leadership through clear AI visions, modelling and targeted training to support the development of nurses’ digital self‐efficacy [18]. Simultaneously, organisations may benefit from efforts to increase perceived job autonomy by granting greater clinical decision latitude and reducing unnecessary micromanagement. These two levers, leadership and autonomy, may work synergistically to reinforce the pathway from leadership support to self‐efficacy and, ultimately, to more effective GenAI collaboration, with potential downstream benefits for workflow efficiency, ethical oversight and workforce resilience in high‐pressure tertiary settings [2].

These strategies support a shift from top–down AI implementation towards distributed, adaptive models that position nurses as active partners with GenAI [24]. Such approaches may support improvements in workflow efficiency, ethical oversight (e.g., critical evaluation of GenAI outputs) and resilience against workload pressures, turnover and rational resistance, thereby helping to address key workforce challenges [18].

### 6.3. Limitations and Future Research

Several limitations should be noted alongside corresponding avenues for future research. First, although a time‐lagged design was used to reduce CMV, head nurse digital leadership was assessed solely through subordinate ratings provided by the staff nurses themselves. This approach, while standard in leadership research [50, 51], may introduce upward bias or halo effects, whereby nurses with higher digital self‐efficacy perceive their leaders as more digitally supportive. To address this, future research should employ multisource data, such as objective GenAI usage logs or self‐reported head nurse ratings, combined with longitudinal designs to examine the long‐term sustainability of these relationships and minimise single‐source evaluation artefacts.

Second, our reliance on nonprobability purposive and convenience sampling within selected nursing departments introduces selection biases that constrain the external generalisability of our findings. Convenience sampling can inadvertently over‐represent frontline nurses who are more technologically accessible or digitally enthusiastic, which may upwardly skew the observed levels of digital self‐efficacy and collaboration. Furthermore, because the sample was drawn exclusively from premier tertiary hospitals in China, the findings may not fully represent smaller, resource‐constrained primary or secondary care facilities, or different cultural contexts. Tertiary facilities typically feature advanced IT infrastructures and structured training programmes that are rarely available in lower tier institutions. Consequently, future comparative studies across different countries, multitiered hospital systems or rural clinics are needed to test how institutional resource variances and cultural moderators, such as high‐power distance in China versus flatter structures elsewhere, affect the model.

Third, while established scales were adapted for GenAI relevance, the custom modifications to the collaboration items warrant further validation in more diverse samples. Qualitative or mixed‐methods approaches could enrich this measurement foundation by offering a deeper understanding of how nurses experience GenAI collaboration under varying autonomy levels. Similarly, intervention studies testing specific leadership training programmes designed to enhance digital leadership and operational autonomy could provide actionable, field‐tested evidence for sustainable AI integration in nursing.

Finally, although the time‐lagged design separated leadership and autonomy at Wave 1 from self‐efficacy and collaboration at Wave 2, the mediator and outcome were assessed concurrently. True longitudinal mediation requires a three‐wave design. Therefore, while the results are consistent with the proposed causal sequence, reverse causality or reciprocal effects cannot be entirely ruled out. Future studies should implement rigorous three‐wave longitudinal frameworks to capture actual causal changes over time. Additionally, configurational approaches, such as fuzzy‐set qualitative comparative analysis (QCA), could expand upon this timeline. These methods could identify the equifinal pathways through which complex combinations of digital leadership, self‐efficacy and autonomy lead to high or low GenAI collaboration, thereby complementing the symmetric net‐effects approach used in this study [52–57].

## Author Contributions

Qian Wu is the sole author of this manuscript and was individually responsible for all aspects of the study, including conceptualisation, methodology, data collection, statistical analysis, formal interpretation and the drafting and revision of the manuscript. The author reviewed and edited the output as needed and takes full responsibility for the final content of the article.

## Funding

The author has nothing to report.

## Ethics Statement

This study received ethical approval from the Ethics Committee of the Department of Nursing at North Anhui Health Vocational College, Suzhou, Anhui, China (No. 20520614).

## Conflicts of Interest

The author declares no conflicts of interest.

## Data Availability

Data can be made available upon reasonable request.
